# Teledermatology and Teledermoscopy for Melanoma Care Pathways: Timeliness, Diagnostic Performance, and Stage at Diagnosis: A Systematic Review

**DOI:** 10.3390/diagnostics15233003

**Published:** 2025-11-26

**Authors:** Alexandra Laura Mederle, Roxana Manuela Fericean, Roxana Grigore, Mihail-Alexandru Badea, Monica Angela Bara, Adrian Cosmin Ilie

**Affiliations:** 1 Department XIV, Discipline of Dermatology, “Victor Babes” University of Medicine and Pharmacy, 300041 Timisoara, Romania; alexandra.mederle@umft.ro (A.L.M.); manuela.fericean@umft.ro (R.M.F.); 2 Doctoral School, “Victor Babes” University of Medicine and Pharmacy, 300041 Timisoara, Romania; 3 Dermatology Department, The George Emil Palade University of Medicine, Pharmacy, Science, and Technology, 540139 Targu Mures, Romania; 4 Faculty of Medicine, “December 1 1918” University, 510009 Alba Iulia, Romania; 5 Department III Functional Sciences, Division of Public Health and Management, Faculty of Medicine, “Victor Babes” University of Medicine and Pharmacy, 300041 Timisoara, Romania; ilie.adrian@umft.ro

**Keywords:** melanoma/diagnosis, telemedicine/methods, dermoscopy/methods, early diagnosis, time-to-treatment

## Abstract

**Background/Objectives**: Early melanoma management depends on rapid triage and definitive treatment. Image-enabled teledermatology, particularly teledermoscopy, may accelerate pathways without compromising safety. This review evaluated effects on timeliness, diagnostic performance, and initial prognostic severity at diagnosis. **Methods**: Following a preregistered PRISMA-2020 protocol, PubMed/MEDLINE, Embase, and Scopus were searched from inception to 1 September 2025. Comparative clinical studies and service evaluations reporting melanoma-specific outcomes were eligible. Dual screening, duplicate data extraction, and risk-of-bias appraisal were performed; substantial clinical and methodological heterogeneity precluded quantitative meta-analysis, so findings were synthesized narratively. **Results:** Twelve studies met criteria. Front-end intervals consistently shortened: time to expert advice was ≈1 day and median time to first clinic was reduced by ~10 days versus conventional referral. Downstream milestones showed mixed translation, with time to excision and histology in several programs remaining ≈45–67 days, indicating capacity constraints beyond triage. Diagnostic safety was high where quantified: melanoma triage sensitivity approached ~99% with a false-negative rate near ~1.1%, and positive predictive value for suspected-melanoma referrals was ~45–50% in mature services. Selected observational comparisons suggested a ≈0.6 mm lower mean Breslow thickness and ~13-percentage-point higher in situ/T1 proportion in one TD network, although these findings are susceptible to selection and lead-time bias and are not consistent across all settings. Overall, teledermoscopy functioned as a “front-door accelerator,” with pathway gains being largest before surgical and pathology steps. **Conclusions:** Teledermatology/teledermoscopy reliably compresses referral-to-decision intervals and maintains high triage safety, with indications of stage migration toward thinner tumors at diagnosis. Integrating surgical and pathology capacity is essential to convert early advice into earlier definitive treatment.

## 1. Introduction

Melanoma outcomes hinge on early detection because prognosis is tightly coupled to pathological stage and Breslow thickness at diagnosis. The AJCC 8th edition formalized this with evidence-based stage refinements and risk stratification that directly translate thickness, ulceration, and nodal status into survival differences relevant to both patients and planners [1,2]. Beyond thickness and AJCC stage, molecular alterations (e.g., BRAF/NRAS mutations, gene-expression signatures) and features of the host immune response (such as tumor-infiltrating lymphocytes) further stratify risk and inform systemic therapy decisions; although, these factors are rarely available at the point of initial referral and triage. European incidence has risen over recent decades, with aging populations, intermittent high-intensity UV exposure, improved ascertainment, and broader behavioral and socioeconomic factors—such as skin-phototype distribution, sun-seeking and tanning behaviors, occupational and recreational exposures, and variable uptake of opportunistic screening—driving a steady case load that challenges specialist capacity; GLOBOCAN 2020 estimates underscore melanoma’s disproportionate mortality burden relative to its incidence share [3]. Against this backdrop, service models that can accelerate evaluation of suspicious lesions—without compromising diagnostic safety—are public-health priorities.

Across many health systems, suspected-cancer pathways aim to compress time from referral to specialist assessment and from decision-to-treat to treatment. Within the United Kingdom, for example, NICE NG12 specifies features warranting urgent referral from primary care to dermatology [4], and national cancer-waiting-time standards articulate a 62-day referral-to-treatment benchmark and a 31-day decision-to-treatment benchmark, with an additional 28-day ‘Faster Diagnosis Standard’ for time to rule-in/rule-out cancer [5]. These UK standards are not uniformly replicated across Europe but serve as illustrative time-bound targets when considering pathway performance. These policy anchors are useful comparators when judging whether novel triage tools such as teledermatology (TD) and teledermoscopy (TDS) deliver pathway-level benefits that matter to patients (earlier decisions) and systems (on-target throughput).

Why speed matters is quantifiable. A large meta-analysis across multiple cancers estimates that each four-week delay in curative-intent treatment increases mortality risk, highlighting the population-level stakes of reducing system delays [6]. Melanoma-specific datasets point the same way: analyses of national and state cohorts associate longer surgical intervals with worse survival, particularly when treatment extends beyond commonly cited windows (e.g., >6–8 weeks or >90 days) [7,8]. While confounding and selection must be considered in observational designs, the consistency of directionality supports timeliness as a legitimate quality domain for melanoma pathways and a pertinent endpoint for TD/TDS evaluations.

Teledermatology’s diagnostic performance for skin cancer has been synthesized across pre-pandemic and contemporary eras. Earlier systematic reviews reported variable but generally high agreement with in-person care for cancer triage, while acknowledging heterogeneity in study quality and reference standards [9,10]. Post-pandemic updates continue to report acceptable accuracy for pigmented lesion triage with store-and-forward and hybrid models, though reliability varies by image quality, lesion selection, and workflow integration—reinforcing the need for robust implementation studies and standardized reporting [11,12]. These syntheses are limited by substantial heterogeneity in design and reference standards, selective publication of better-performing implementations, and the fact that many studies did not apply histopathology to all triaged lesions, which may overestimate accuracy. Practical reviews and focused analyses of neoplasm triage suggest accuracy around the 75–80% range in many programs, approaching face-to-face benchmarks when dermoscopy and structured protocols are used [13]. Framing TD/TDS against pathway targets (faster-diagnosis and 62-day standards) clarifies its role as a front-end accelerator rather than a universal substitute for dermato-oncology examination and biopsy.

Pandemic disruptions provided a stress test of pathway resilience. Across Europe, service interruptions and reduced face-to-face access were associated with fewer melanoma diagnoses during lockdowns, followed by a rebound enriched for thicker tumors and adverse features; modeling suggests substantial years-of-life lost and macroeconomic costs linked to these delays [14,15]. These findings sharpen our study focus: the value proposition of TD/TDS is not generic convenience but targeted pathway decongestion that preserves diagnostic safety while pulling forward the moment of decision for lesions at genuine risk of invasion.

Accordingly, the current study aims to address the following melanoma-related issues: (i) timeliness (referral-to-dermatologist and time-to-excision), (ii) diagnostic performance (sensitivity/specificity, PPV, false-negative rates) using appropriate reference standards, and (iii) initial prognosis at diagnosis (Breslow thickness and stage). By mapping these outcomes to pathway targets and staging-anchored risk, we aim to identify when and how TD/TDS improves patient-important outcomes, where reporting needs standardization, and what policy levers can translate evidence into scalable cancer-pathway design.

## 2. Materials and Methods

### 2.1. Protocol and Registration

This review was planned, conducted, and reported according to PRISMA-2020 guidance [16]. The protocol prespecified the review questions, eligibility criteria, outcomes, data extraction fields, and bias-appraisal tools, and was prospectively registered on the Open Science Framework (OSF code osf.io/kbpt6). Protocol fidelity was verified by a second reviewer who was not involved in initial screening, through audit of the final eligibility criteria, search strategies, and extracted data fields against the OSF-registered protocol and by checking that no post hoc changes were introduced that would alter study selection or primary outcomes.

### 2.2. Review Question and Framework

The review asked whether image-enabled teledermatology, including teledermoscopy, for patients referred with suspicious cutaneous lesions improves pathway timeliness, diagnostic performance, and initial prognostic severity at diagnosis compared with conventional non-image-based referral. The population comprised individuals of any age evaluated in primary care, urgent cancer pathways, or mixed dermatology referral streams where melanoma outcomes were reported at the study or subgroup level. The intervention was asynchronous or synchronous teledermatology using clinical and/or dermoscopic images captured in the community or clinic, including smartphone workflows, virtual lesion clinics, and regional or national teledermoscopy programs. Comparators were standard paper or electronic referrals without images, face-to-face first assessments, or pre/post implementation periods.

Because the overarching policy question concerned whether TD/TDS can safely ‘pull forward’ decisions along melanoma pathways, pathway timeliness (days to first specialist decision, biopsy or excision, and histopathology report) and diagnostic performance (sensitivity, specificity, positive predictive value, number-needed-to-excise, and false-negative rate) were prespecified as co-primary outcome domains when an appropriate reference standard was used. Initial prognosis at diagnosis (Breslow thickness in millimeters, ulceration, and AJCC 8th-edition stage distribution) was treated as a secondary mechanistic domain, used to explore potential stage-related consequences of earlier triage.

### 2.3. Eligibility Criteria

Eligible designs were randomized trials; prospective or retrospective comparative cohorts; controlled before–after evaluations; service evaluations with extractable arm-level or patient-level data for at least one prespecified primary outcome; and diagnostic accuracy or triage studies using verification by histopathology or structured follow-up. Ineligible records were case reports or very small series with fewer than ten melanoma cases; editorials, letters, narrative reviews, or technical validation without clinical pathway endpoints; pure algorithm papers without human clinical decision-making; and non-human research. No limits by language, geography, or publication year were applied at search stage. Studies that reported mixed “skin cancer” cohorts were included only when melanoma-specific results were extractable or verifiably incorporated into the endpoint.

### 2.4. Information Sources and Dates

We interrogated PubMed/MEDLINE, Embase (Elsevier), and Scopus (Elsevier) from database inception through 1 September 2025. Syntax and controlled vocabulary were translated across databases using validated filters where applicable. Searches were last executed on 1 September 2025.

PubMed/MEDLINE (inception to 1 September 2025). (“Melanoma”[Mesh] OR melanoma*[tiab] OR “skin neoplasm*”[tiab] OR “pigmented lesion*”[tiab]) AND (teledermatolog*[tiab] OR “tele-dermatolog*”[tiab] OR teledermoscop*[tiab] OR “tele-dermoscop*”[tiab] OR ((dermoscop*[tiab] OR “dermoscopy”[Mesh]) AND (telemedicin*[tiab] OR smartphone*[tiab] OR mobile[tiab] OR “store-and-forward”[tiab] OR asynchronous[tiab] OR virtual[tiab] OR “two-week wait”[tiab] OR 2WW[tiab] OR “urgent cancer”[tiab]))).

Embase (Elsevier; incept–1 September 2025; Emtree mapped). (‘melanoma’/exp OR melanoma*:ti,ab OR ‘skin neoplasm*’:ti,ab OR ‘pigmented lesion*’:ti,ab) AND (teledermatology:ti,ab OR ‘tele-dermatology’:ti,ab OR teledermoscopy:ti,ab OR ‘tele-dermoscopy’:ti,ab OR (dermoscopy:ti,ab OR ‘dermoscopy’/exp) AND (telemedicine:ti,ab OR ‘telemedicine’/exp OR smartphone*:ti,ab OR ‘smartphone’/exp OR mobile:ti,ab OR ‘store and forward’:ti,ab OR asynchronous:ti,ab OR virtual:ti,ab OR ‘two week wait’:ti,ab OR 2ww:ti,ab OR ‘urgent cancer’:ti,ab)).

Scopus (Elsevier; incept–1 September 2025; TITLE-ABS-KEY). TITLE-ABS-KEY(melanoma* OR “skin neoplasm*” OR “pigmented lesion*”) AND TITLE-ABS-KEY(teledermatolog* OR tele-dermatolog* OR teledermoscop* OR tele-dermoscop* OR (dermoscop* AND (telemedicin* OR smartphone* OR mobile OR “store-and-forward” OR asynchronous OR virtual OR “two-week wait” OR 2WW OR “urgent cancer”))).

### 2.5. Study Selection

**Figure 1:**
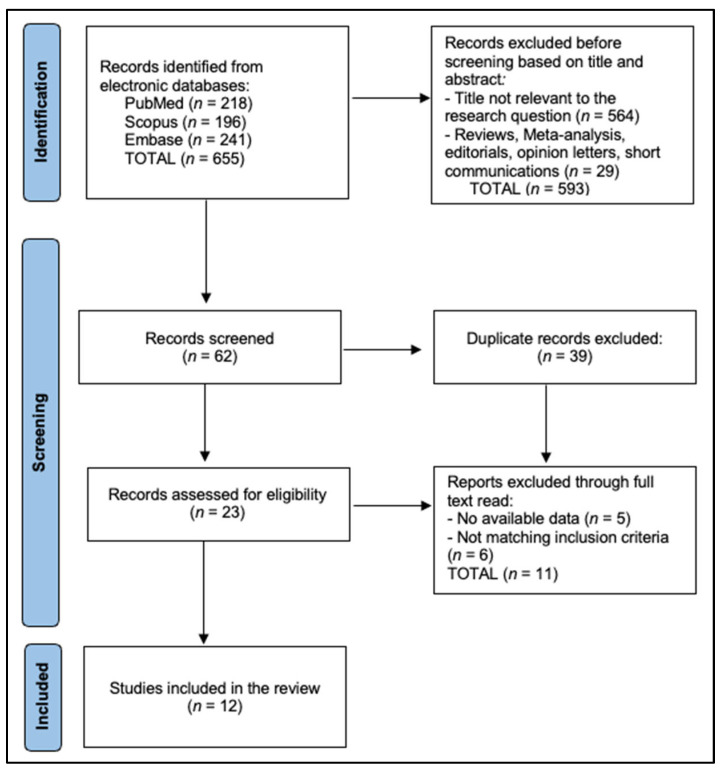
PRISMA Flowchart.

Records were exported to EndNote X20 for deduplication and then into Rayyan QCRI for blinded dual screening. Two reviewers independently screened titles and abstracts against a piloted form, resolving disagreements by consensus or third-party adjudication. Calibration on a 10% random subset achieved a Cohen’s κ of at least 0.80 for inclusion decisions, after which the screening form was locked. The PRISMA flow is as follows: 655 records were identified across databases (PubMed 218, Embase 241, Scopus 196). After deduplication of 39 records, 616 unique records underwent title and abstract screening, of which 593 were excluded as irrelevant by condition, intervention, or article type. Twenty-three full texts were assessed for eligibility; eleven were excluded due to absent melanoma-specific extractable data in mixed cohorts (*n* = 5) or ineligible design/setting without pathway endpoints (*n* = 6). Mixed ‘skin cancer’ cohorts without extractable melanoma-specific outcomes were excluded to avoid misattributing non-melanoma endpoints (e.g., basal cell or squamous cell carcinoma) to melanoma. This approach inevitably favors settings with more granular diagnostic coding and reporting; as a result, our included sample may under-represent programs where melanoma outcomes are embedded in broader skin cancer metrics. Twelve studies met all criteria and were included in the narrative synthesis (Figure 1).

### 2.6. Risk-of-Bias Assessment and Synthesis

Risk of bias was appraised independently by two reviewers at the outcome level using RoB 2 for randomized trials, ROBINS-I for non-randomized comparative designs and service evaluations, and QUADAS-2 for diagnostic accuracy and triage studies. Prespecified confounders for ROBINS-I included secular trends, concurrent pathway reconfiguration, differential imaging quality, and variation in verification intensity. Any discrepancies were resolved by consensus.

Given anticipated clinical and methodological heterogeneity, we prespecified a structured narrative synthesis. For each study, we extracted the operational definitions used for timeliness (referral-to-first specialist contact vs. referral-to-biopsy), diagnostic performance (per-lesion vs. per-patient denominators, inclusion of benign nevi), and prognostic severity (Breslow thickness distribution, AJCC stage). These definitions were tabulated and compared qualitatively. Because of non-overlapping time points, differing denominators, and variable reference standards, we did not perform meta-analytic pooling; instead, we focused on the direction and consistency of effects within outcome domains.

Risk-of-bias assessments were performed for all twelve included studies [17,18,19,20,21,22,23,24,25,26,27,28], as summarized in Table 1, Table 2 and Table 3. The single randomized trial [27] was judged to have some concerns due to limited information on allocation concealment and small sample size. Most non-randomized comparative and service-evaluation studies were at serious risk of bias for confounding under ROBINS-I, reflecting before–after designs without formal adjustment for secular trends, pathway reconfiguration, or changes in referral behavior. Selection of participants into TD versus conventional pathways was frequently clinician- or patient-driven rather than random. Outcome measurement (Breslow thickness, time stamps) was generally at low risk of bias, but diagnostic-accuracy studies commonly had unclear or high risk in QUADAS-2 verification domains because not all triaged lesions underwent histopathology. These limitations constrain causal inference, particularly for prognostic endpoints.

For ROBINS-I, prespecified confounders included secular trends in melanoma incidence and pathway performance, concurrent organizational changes (e.g., introduction of faster-diagnosis standards or additional theatre lists), case-mix differences between teledermatology and conventional streams, and differential verification intensity. During risk-of-bias assessment, we recorded whether studies reported adjustment for these factors (multivariable regression, difference-in-differences analyses, or stratified reporting); none fully accounted for all prespecified confounders. Risk of bias is presented in Table 1, Table 2 and Table 3.

We explored the feasibility of pooling melanoma triage sensitivity and positive predictive value (PPV) but found that only two real-world programs reported numerators and denominators in a sufficiently comparable fashion, with differences in inclusion of in situ disease and verification strategies. A meta-analysis based on such sparse and heterogeneous data was deemed unlikely to yield a meaningful pooled estimate, and results are therefore presented descriptively.

## 3. Results

Across 12 melanoma-relevant evaluations, settings ranged from single-service pilots to national programs, with several providing sizable melanoma samples. The 12 evaluations comprised one randomized controlled trial [27], several prospective comparative or before–after cohorts [20,25], diagnostic-agreement and cross-sectional studies [21,28], and multiple retrospective cohorts and service evaluations [17,18,19,22,23,24,26], reflecting the predominantly implementation-oriented nature of the evidence base. A Spanish retrospective cohort focused exclusively on melanoma (*n* = 201) compared store-and-forward teledermatology (TD) with conventional referral and prespecified prognosis endpoints [17]. Early feasibility and workflow papers established smartphone teledermoscopy (TDS) in Sweden and a Virtual Lesion Clinic (VLC) in New Zealand for mixed referrals that included melanoma subsets [18,19]. In Sweden, countywide TDS correctly handled 94/95 melanomas (sensitivity 98.9%; false-negative rate 1.10%) over the observed follow-up [28], and a mature Virtual Lesion Clinic in New Zealand reported a similarly low false-negative rate (~1.2%) in a high-throughput triage context [22]. However, neither program provided long-term linkage to subsequent advanced melanoma diagnoses among initial false negatives.

A UK prospective comparative study examined time to clinic within an urgent cancer pathway [20], while a Swedish agreement study assessed diagnostic concordance and direct-to-surgery booking using TDS referrals versus paper referrals [21]. Service-level triage performance was reported from a mature VLC (PPV/FNR endpoints) [22], and a retrospective review from New Zealand quantified throughput (402 referrals; 19 melanomas) and time-to-advice [23]. A country-wide Estonian TDS program contributed 4748 cases with diagnostic and plan accuracy benchmarking [24]. A UK two-week wait (2WW) evaluation compared virtual versus face-to-face outcomes [25]. A U.S. institutional comparison contrasted TD-first versus in-person-first melanoma pathways with head-to-head clinical endpoints [26]. A French randomized trial studied smartphone photo relay from primary care and its effect on time to dermatology consult [27], and a Swedish countywide analysis reported melanoma triage sensitivity in a real-world TDS rollout (*n* = 135 melanomas; 95 via TDS) [28], as presented in Table 4.

The included evaluations spanned heterogeneous designs and settings, ranging from single-center pilots and feasibility studies to regional and national services. Operational definitions for timeliness varied (time from referral to first dermatology contact, time to biopsy, or time to histology report), as did the staging metrics (Breslow thickness as a continuous variable, dichotomous in situ/T1 vs. >T1, or AJCC stage categories). Diagnostic performance was variously reported per lesion or per patient, and verification strategies ranged from systematic histopathology to selective biopsy with clinical follow-up. This heterogeneity precluded meaningful quantitative pooling and informed our choice of a narrative synthesis.

In Spain, a regional store-and-forward TD network was associated with a lower mean Breslow thickness (1.06 mm vs. 1.64 mm) and a higher proportion of in situ/T1 melanomas (70.1% vs. 56.9%) in the TD arm compared with conventional referral [17]. In contrast, the U.S. institutional cohort by Jaklitsch et al. [26] reported that melanomas detected through TD-first pathways were, on average, thicker and more often ulcerated than those detected through in-person-first pathways, reflecting selective channeling of more clinically worrisome lesions into TD rather than a simple, uniform ‘thinning’ effect across all settings.

TD/TDS compressed early pathway intervals and, in some contexts, was associated with more favorable staging at diagnosis. In the UK, photo-triage halved the median time to clinic (14 vs. 24 days, melanoma subset) without breaching the 62-day standard for nearly all patients [20]. In Spain, TD was associated with a lower mean Breslow thickness (1.06 mm vs. 1.64 mm) and a higher proportion of in situ/T1 melanomas (70.1% vs. 56.9%) compared with conventional referral [17]. Operationally, a New Zealand review reported dermatologist advice at ≈1.02 days but a downstream “time to action” around 64.8 days, highlighting post-triage capacity constraints [23]. A French RCT showed a shorter delay to dermatology consultation when GPs relayed smartphone photos versus usual scheduling [27]. In a U.S. cohort, TD-first accelerated evaluation and biopsy (both *p* < 0.001), although TD-first melanomas presented thicker and more often ulcerated—consistent with selection of higher-risk lesions into the TD-first stream [26], as seen in Table 5.

In Sweden, countywide TDS correctly handled 94/95 melanomas (sensitivity 98.9%) with a 1.10% false-negative rate, supporting safe large-scale triage [28]. A mature VLC in New Zealand reported an overall PPV ≈49% for suspected melanoma referrals with an ≈1.2% false-negative rate—acceptable in a high-throughput triage context [22]. A Swedish agreement study observed higher diagnostic concordance and more direct-to-surgery bookings with TDS referrals than with paper-based referrals, indicating improved operational precision [21]. Estonia’s national TDS service (4748 cases) showed diagnostic and management plan accuracy comparable to experimental settings, suggesting strong external validity at scale [24]. Additional service reports underscored speed-of-advice (~1 day) with relatively few melanomas within total referrals [23], maintenance of 2WW breach times with a notable 42.9% increase in SCC detection under virtual pre-assessment (*p* = 0.03) [25], and feasibility signals from early smartphone-based TDS that later informed pathway design [18] (Table 6).

UK photo-triage reduced median time to clinic to 14 days versus 24 days while meeting 62-day treatment targets [20]. In Spain, a regional TD network showed thinner melanomas and a higher in situ/T1 share in the TD arm [17]. Swedish pilots demonstrated that GP-captured smartphone dermoscopy could return expert reads often within 24 h and supported multicenter scaling [18,21]. New Zealand VLC models typically delivered advice in ~1 day with moderate PPV and low FNR, reflecting robust triage but revealing downstream capacity as the main bottleneck [19,22,23]. A UK 2WW virtual pathway maintained breach times and improved SCC detection [25]. Estonia’s nationwide TDS embedded 1–2 day dermatologist turnaround within the e-health infrastructure and reproduced experimental-level diagnostic/plan accuracy at national scale [24]. A French RCT confirmed faster access to dermatology consultation via smartphone photo relay from primary care [27], and a U.S. academic system showed TD-first accelerated evaluation and biopsy while triaging a clinically higher-risk case-mix [26]. A Swedish countywide rollout documented triage sensitivity of 98.9% for melanoma, reinforcing safety at scale [28] (Table 7).

**Figure 2:**
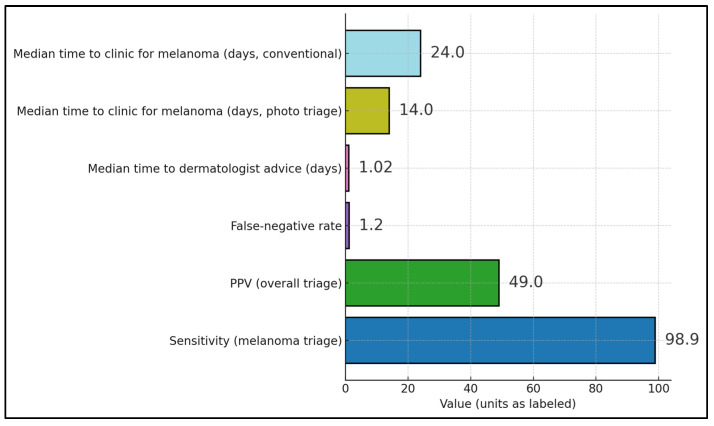
Reported metrics across the included studies.

Triage safety was high where quantified, with melanoma sensitivity of 98.9% in a county-wide teledermoscopy (TDS) rollout (false-negative rate ≈ 1.1%) in Sweden [28], while a mature Virtual Lesion Clinic reported PPV ≈ 49% and FNR ≈ 1.2% for suspected-melanoma triage [22]. Operational speed at the front end was rapid: median time to dermatologist advice via store-and-forward teledermatology was ~1.02 days in a New Zealand service [23]. For pathway timeliness, a UK prospective study showed a median time to first clinic of 14 days with photo-triage versus 24 days via conventional urgent referral, illustrating upstream acceleration with TD/TDS [20], as presented in Figure 2.

**Figure 3:**
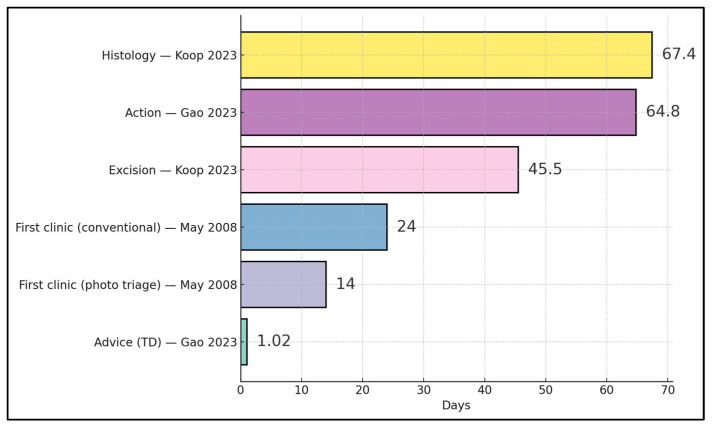
Timeline milestones across TD/TDS pathways (days) [20,23,24].

Front-end advice occurred at ~1.02 days via TD in New Zealand [23], and photo-triage halved time to first clinic (14 vs. 24 days) in the UK [20]. In contrast, later milestones were longer: a national-scale Estonian program reported median ~45.5 days to excision and ~67.4 days to histology for triaged lesions [24], while a New Zealand service observed ~64.8 days to “time to action,” indicating that gains from rapid triage can be attenuated by surgical and histopathology throughput constraints downstream [23,24] (Figure 3).

Across the 12 studies, reporting of equity-relevant variables was limited. Few evaluations provided outcomes stratified by socioeconomic status, ethnicity, rural versus urban residence, or digital literacy, and none reported triage performance separately in these strata. Lesion-level detail (anatomic site, histologic subtype) was also variably reported, limiting subgroup analyses.

## 4. Discussion

### 4.1. Summary of Evidence

Teledermatology (TD) and teledermoscopy (TDS) are best positioned as front-end accelerators of melanoma pathways, compressing referral-to-dermatologist intervals and facilitating earlier biopsy without replacing definitive face-to-face dermato-oncology. Evidence syntheses and guidance consistently frame TD/TDS as structured triage that “pulls forward” the diagnostic decision when dermoscopic images are captured at the first point of contact and routed via standardized workflows [29,30].

Stage-related signals observed in selected programs using image-enabled triage—most notably the lower mean Breslow thickness and higher in situ/T1 proportion in the Spanish TD cohort [17]—suggest that teledermatology can be associated with detection of thinner melanomas in some contexts. However, these findings arise from a small number of observational comparisons with unmeasured confounding, and they contrast with the U.S. TD-first cohort by Jaklitsch et al. [26], which was enriched for thicker, more ulcerated tumors. Taken together, the evidence for a consistent ‘stage shift’ remains low-certainty and context-dependent [31,32,33,34]. The U.S. TD-first experience [26] highlights that teledermatology can be preferentially used for higher-risk lesions, leading to an apparent enrichment for thicker, ulcerated melanomas in TD streams. Rather than contradicting earlier detection in other programs, these findings underscore that case-selection and implementation context strongly shape observed stage distributions and must be considered when interpreting any apparent ‘stage shift’.

Diagnostic safety is central to scaled triage. Contemporary real-world accuracy studies indicate that TDS achieves substantial agreement with reference diagnoses when image quality and lesion selection are controlled; risks concentrate in lower-quality images and ambiguous morphologies, supporting capture standards and escalation rules [35]. National-scale experience further suggests that diagnostic and management accuracy achieved in experimental settings can transfer to routine services when protocols and turnaround expectations are codified [24,36]. Across studies, however, the processes for ensuring adequate image quality and appropriate lesion selection were heterogeneous and often poorly described, underscoring the need for validated, reproducible quality-control criteria, and training frameworks to support safe scaling.

Operationally, TD/TDS reliably reduces time to expert advice, yet translation into shorter time to definitive treatment depends on synchronized capacity downstream (theater, pathology, multidisciplinary meetings). Health-economic evaluations generally find store-and-forward teledermatology to be cost-saving or cost-effective as a triage mechanism—particularly where travel distances are large or specialist access is sparse—while emphasizing that pathway-level integration is necessary to realize those gains [37,38]. Broader digital-dermatology appraisals highlight organizational readiness, workflow integration, and equity of digital access as key moderators of realized benefit [39]. In the Estonian national program, for example, dermatologist reads were typically available within 1–2 days, yet median intervals to excision and histology remained around 45.5 and 67.4 days, respectively [24]. Similarly, in the New Zealand chart review, advice was returned at ≈1 day while ‘time to action’ extended to ≈65 days [23]. Authors of these evaluations attributed the residual delays largely to operating-theater scheduling, surgical workforce constraints, and histopathology laboratory throughput rather than to the teledermatology step itself, although explicit queue-time audits were uncommon. These observations suggest that front-end acceleration must be coupled with co-planned theater sessions, ring-fenced cancer lists, and aligned histopathology reporting targets if earlier triage is to translate into earlier definitive treatment.

Implementation quality remains the principal determinant of performance. Guidance reviews and expert consensus statements recommend routine reporting of core variables (lesion spectrum, reference standard, dermoscopic image quality metadata), training for dermoscopic capture in primary care, second-reader safeguards for equivocal lesions, and explicit safety-netting triggers for in-person review [29,30]. Notably, none of the primary studies included in this systematic review formally evaluated second-reader safeguards (for example, mandatory secondary review of equivocal lesions) as a distinct intervention component; this recommendation therefore rests on external expert guidance and broader teledermatology safety principles rather than direct comparative evidence within our dataset. Programs that embed these features at the regional or national scale demonstrate high throughput with sustained diagnostic safety, while also revealing equity considerations—such as variable feasibility of high-quality image capture across primary-care settings—that warrant targeted support [24,39].

Therefore, a pragmatic policy approach is to scale TDS at the referral front door; require minimum dermoscopy-quality standards; and explicitly co-plan downstream capacity—for example, linking TD/TDS slots to dedicated cancer theater lists, protected biopsy clinics, and histopathology reporting benchmarks that are compatible with national faster-diagnosis targets.

Equity considerations are particularly important for TD/TDS, which require access to imaging devices, connectivity, and digital literacy. The studies included in this review were largely conducted in high-income health systems and rarely reported outcomes by age, socioeconomic position, or ethnicity. Outside our dataset, observational work during the COVID-19 era has shown that older adults and socioeconomically disadvantaged populations are less likely to complete video or teledermatology visits despite similar clinical need, illustrating a persistent ‘digital divide’ [38,39]. Ensuring that TD/TDS does not exacerbate existing disparities will require targeted support for image capture in under-resourced primary-care settings, accessible technical pathways for patients with limited digital skills, and routine equity-focused monitoring. Readiness and safety thresholds for wider implementation are likely to vary across health systems and should be grounded in local pilot data demonstrating acceptable timeliness, diagnostic performance, and safety-netting, rather than assumed from external benchmarks alone.

### 4.2. Limitations

The evidence base remains heterogeneous in design, settings, and verification intensity, limiting meta-analytic pooling. In particular, most comparative studies were observational and did not include robust adjustment for secular trends, evolving referral criteria, or differences in lesion case-mix between TD/TDS and conventional pathways. As a result, associations between teledermatology exposure and either timeliness or Breslow thickness may be partly driven by channeling of more complex or more suspicious cases into specific pathways rather than by the triage modality itself. Second, apparent ‘stage shift’ toward thinner tumors in some cohorts may reflect lead-time bias, whereby earlier detection identifies lesions at an earlier point in their natural history without necessarily changing ultimate outcomes. None of the included studies were designed to link TD/TDS exposure to long-term survival, recurrence, or late presentation of initially missed lesions. As a result, both the safety profile and prognostic impact of TD/TDS must be interpreted with caution and validated in longitudinal datasets. Third, our search was limited to PubMed/MEDLINE, Embase, and Scopus and did not include trial registries, CENTRAL, or gray-literature repositories. Ongoing or locally reported teledermatology evaluations may therefore have been missed, and future reviews should extend coverage to these sources. Fourth, although several programs reported diagnostic performance metrics (e.g., sensitivity, PPV, false-negative rate), only a minority did so using comparable denominators and thresholds. This precluded formal meta-analytic pooling of accuracy estimates, and our synthesis of diagnostic performance is therefore descriptive rather than quantitative.

Several evaluations were single-center service studies with potential selection bias and confounding from concurrent pathway reconfiguration. Reporting of core variables (image quality metrics, reference standards, per-lesion vs. per-patient denominators) was inconsistent, and downstream outcomes (time to excision, histology) were often influenced by local capacity rather than teledermatology itself. Cost and equity data were sparse, and few studies reported standardized safety-net protocols or second-reader safeguards. The often-quoted estimate that each four-week delay in curative-intent treatment is associated with increased mortality risk derives from a mixed-cancer meta-analysis largely focused on internal organ malignancies [6]. In this review we use it as conceptual support for the importance of timeliness rather than as a melanoma-specific quantitative benchmark. Taken together, these limitations mean that our strongest conclusions pertain to early pathway timeliness and short-term triage performance, outcomes that were relatively consistently defined and measured. In contrast, inferences about prognostic impact, cost-effectiveness, and equity are necessarily more tentative: they rely on fewer studies, with greater risk of confounding and sparse reporting of relevant covariates. We therefore regard our statements on stage distribution, economic value, and equity as hypothesis-generating rather than definitive.

Future studies should (i) report standardized, melanoma-specific outcome sets encompassing timeliness, diagnostic accuracy, and stage at diagnosis; (ii) incorporate equity-sensitive analyses across age, socioeconomic position, and geography; (iii) evaluate the incremental value and cost-effectiveness of TD/TDS using robust economic modeling; and (iv) link teledermatology exposure to long-term survival and recurrence outcomes using high-quality registry or administrative data. Such work will be essential to determine whether the promising pathway gains observed here translate into durable clinical benefit at population scale.

## 5. Conclusions

Image-enabled referral, especially teledermoscopy, appears to be a promising and generally safe mechanism to shorten early melanoma pathway intervals. Limited observational data suggest possible associations with thinner disease at diagnosis in some settings, but the overall certainty regarding a true stage shift is low and alternative explanations, including selection and lead-time bias, cannot be excluded.

## Figures and Tables

**Table 1:** Risk of bias assessment of non-randomized comparative and service-evaluation studies (ROBINS-I).

Study (Year)	Design/Setting	Confounding	Selection of Participants	Classification of Interventions	Deviations from Intended Interventions	Missing Data	Measurement of Outcomes	Selection of Reported Result	Overall Risk of Bias
Ferrándiz 2012 [17]	Retrospective cohort, TD vs. conventional	Serious (no adjustment for secular trends or case–mix)	Moderate (clinic-based sample)	Low (clear TD vs. conventional)	Low (routine care)	Moderate (incomplete documentation possible)	Low (histology, standard staging)	Moderate (limited protocol transparency)	Serious
May 2008 [20]	Prospective comparative cohort, photo-triage vs. urgent pathway	Serious (non-random allocation; no formal adjustment)	Moderate (urgent pathway referrals only)	Low	Low	Low	Low	Moderate	Serious
Börve 2013 [18]	Feasibility, non-comparative mobile TDS in primary care	Critical (no comparator; descriptive feasibility only)	Serious (selected GP practices, early adopters)	Moderate	Low	Moderate	Low (technical/operational outcomes)	Serious (selective reporting likely)	Critical
Congalton 2015 [19]	Virtual Lesion Clinic service evaluation (TDS triage)	Serious (no adjustment for secular trends or referral changes)	Moderate (referred lesions only)	Low	Low	Moderate	Low (histology/clinical follow-up)	Moderate	Serious
Gao 2023 [23]	Retrospective chart review, TD service	Critical (no comparator; confounding not addressed)	Serious (single-center, selected referrals)	Low	Low	Moderate	Low (chart-based outcomes)	Moderate	Critical
Chiru 2022 [25]	Before–after 2WW virtual vs. face-to-face	Serious (before–after design; pandemic effects)	Moderate	Low	Low	Low	Low	Moderate	Serious
Jaklitsch 2024 [26]	Retrospective comparative cohort, TD-first vs. in-person-first	Serious (TD-first stream enriched for high-risk lesions; limited adjustment)	Moderate	Low	Low	Low–Moderate	Low (histology and standard pathology)	Moderate	Serious

ROBINS-I categories: Low, Moderate, Serious, Critical risk of bias.

**Table 2:** Risk of bias assessment of randomized trial (RoB 2).

Study (Year)	Design/Setting	Bias Arising from the Randomization Process	Bias Due to Deviations from Intended Interventions	Bias Due to Missing Outcome Data	Bias in Measurement of the Outcome	Bias in Selection of the Reported Result	Overall Risk of Bias
Bouton 2024 [27]	Randomized trial, GP smartphone photos vs. usual scheduling	Some concerns (limited detail on allocation concealment and sequence generation)	Low (no evidence of differential co-interventions)	Low (minimal loss to follow-up)	Low (time-to-consult from records)	Some concerns (no pre-published statistical analysis plan)	Some concerns

RoB 2 categories: Low risk of bias, Some concerns, High risk of bias.

**Table 3:** Risk of bias assessment of diagnostic accuracy and triage studies (QUADAS-2).

Study (Year)	Design/Setting	Patient Selection (RoB)	Index Test (TD/TDS) (RoB)	Reference Standard (RoB)	Flow and Timing (RoB)	Overall Risk of Bias
Dahlén Gyllencreutz 2017 [21]	Diagnostic agreement study, TDS referrals vs. paper	High (non-consecutive; referral-based)	Low (index test interpreted as in practice)	Unclear–High (not all lesions histologically verified)	High (partial verification; variable follow-up)	High
Teague 2022 [22]	VLC triage system for suspected melanoma	Moderate (structured referrals, but not clearly consecutive)	Low	Unclear–High (selective histology; clinical follow-up for some lesions)	Moderate–High (variable verification and follow-up)	High
Koop 2023 [24]	National TDS database, management and diagnostic accuracy	Moderate (large national cohort; referral-based)	Low	Moderate (mix of histology and clinical follow-up as reference)	Moderate (retrospective verification; some attrition)	Moderate
Zazo 2024 [28]	County-wide TDS triage for melanoma	Low–Moderate (population-based county cohort)	Low	Moderate (not all lesions histologically confirmed)	Moderate (follow-up not uniform for all lesions)	Moderate
Börve 2013 [18]	Feasibility study of mobile TDS app	High (convenience sample of participating GPs)	Low	Unclear (limited description of reference verification)	High (incomplete verification; short follow-up)	High
Congalton 2015 [19]	Virtual Lesion Clinic, melanoma triage	High (subset of referred lesions; not clearly consecutive)	Low	Unclear–High (verification mainly for excised lesions)	High (partial verification and variable follow-up)	High

QUADAS-2 risk-of-bias categories: Low, High, Unclear.

**Table 4:** Included studies and core characteristics.

Study (Year)	Country	Design and Setting	*N/*Melanoma Focus	Pathway/Modality	Comparator	Primary Endpoints
Ferrándiz 2012 [17]	Spain	Retrospective cohort (TD vs. conventional)	*n* = 201 melanomas	Store-and-forward TD network	Conventional referral	Breslow thickness; in situ/superficial proportion
Börve 2013 [18]	Sweden	Descriptive/feasibility (mobile TDS app)	Mixed lesions	Smartphone teledermoscopy	None	Feasibility/technical concept (no melanoma outcomes)
Congalton 2015 [19]	New Zealand	Virtual Lesion Clinic (TDS triage)	Mixed referrals; melanoma subset	Teledermoscopy	Usual referral	Triage success/operational outcomes (melanoma subset)
May 2008 [20]	UK	Prospective comparative cohort	Melanoma + SCC (extractable melanoma time to clinic)	Photo-triage (store-and-forward)	Conventional urgent pathway	Time to clinic; 62-day treatment
Dahlén Gyllencreutz 2017 [21]	Sweden	Diagnostic agreement study	Mixed referrals	Mobile TDS referral	Paper referral (no images)	Agreement, inter-observer concordance, direct-to-surgery bookings
Teague 2022 [22]	New Zealand	Service evaluation (TDS triage)	Suspected melanoma referrals	TDS (Virtual Lesion Clinic)	—	PPV, false-negative rate (FNR), NNE
Gao 2023 [23]	New Zealand	Retrospective chart review	402 referrals (19 melanomas)	Store-and-forward teledermatology	—	Time-to-advice; management outcomes
Koop 2023 [24]	Estonia	National TDS database study	4748 cases	Country-wide teledermoscopy	—	Diagnostic/management accuracy vs. experimental settings
Chiru 2022 [25]	UK	Before/after 2WW virtual consults	2WW pathway (MM, NMSC)	Virtual consultations	Face-to-face	Breach time; detection probabilities
Jaklitsch 2024 [26]	USA	Retrospective comparative	Melanomas detected via TD vs. FTF	Teledermatology first vs. in-person first	Head-to-head	Time-to-evaluation/biopsy; Breslow/ulceration
Bouton 2024 [27]	France	Randomized trial (GP→derm photo transfer)	Suspicious lesions (melanoma subset)	Smartphone photo relay	Usual scheduling	Time to dermatology consultation
Zazo 2024 [28]	Sweden	Retrospective cross-sectional (countywide)	*n* = 135 melanomas (95 via TDS)	Teledermoscopy triage	—	Sensitivity for melanoma triage; safety

FTF—face-to-face; FNR—false-negative rate; MM—malignant melanoma; *N*—sample size; NMSC—non-melanoma skin cancer; NNE—number-needed-to-excise; PPV—positive predictive value; RCT—randomized controlled trial; TD—teledermatology; TDS—teledermoscopy; 2WW—two-week wait (UK urgent cancer pathway).

**Table 5:** Timeline and staging.

Study	Time to Clinic (TD/TDS)	Time to Clinic (Conventional)	Time-to-Biopsy/Excision	Breslow Thickness (TD/TDS)	Breslow (Conventional)	In Situ/Superficial (%)
May 2008 (UK) [20]	Median 14 d (melanoma)	Median 24 d (urgent FTF)	NR	NR	NR	NR (narrative: all but one melanoma treated ≤62 d in photo group)
Ferrándiz 2012 (Spain) [17]	NR	NR	NR	Mean 1.06 mm (TD)	Mean 1.64 mm	Tis/T1: 70.1% (TD) vs. 56.9%
Gao 2023 (NZ) [23]	Dermatologist advice median 1.02 d	—	“Time to action” median ~64.8 d	NR	NR	NR
Bouton 2024 (France) [27]	Shorter time to dermatology consult with smartphone photo transfer (randomized)	Longer time to consult	NR	NR	NR	NR
Jaklitsch 2024 (USA) [26]	Shorter wait to initial evaluation via TD (*p* < 0.001)	Longer	Shorter time to biopsy via TD (*p* < 0.001)	Thicker and more ulcerated in TD-first group (*p* < 0.001; *p* = 0.002)	Thinner	NR

d—days; FTF—face-to-face; NR—not reported; NZ—New Zealand; TD—teledermatology; TDS—teledermoscopy; Tis—melanoma in situ; UK—United Kingdom; USA—United States.

**Table 6:** Diagnostic-triage performance and operational metrics.

Study	Sensitivity (Melanoma)	Specificity/PPV	False-Negative Rate	Operational Notes
Zazo 2024 (Sweden) [28]	98.9% (94/95 correctly handled)	NR	1.10%	TDS used in 70% of melanomas county-wide
Teague 2022 (NZ) [22]	NR	PPV ≈ 49% (overall)	~1.2% FN	Structured triage for suspected melanoma
Dahlén Gyllencreutz 2017 (Sweden) [21]	NR	NR	NR	Higher diagnostic agreement, more malignant lesions booked direct-to-surgery vs. paper referral
Koop 2023 (Estonia) [24]	NR	Diagnostic and plan accuracy comparable to experimental settings	NR	4748 cases; national service
Congalton 2015 (NZ) [19]	NR	NR	NR	Successful teledermoscopy triage in virtual clinic
Gao 2023 (NZ) [23]	NR	NR	NR	Advice ~1 day; 19/402 melanomas
Chiru 2022 (UK) [25]	NR	“Equivalent outcomes” MM FTF vs. virtual; SCC detection ↑42.9% with virtual (*p* = 0.03)	NR	2WW breach time 12 d (IQR 6) both arms
Börve 2013 (Sweden) [18]	NR	NR	NR	App feasibility; catalyzed later TDS pathways

FN—false negative; IQR—interquartile range; NR—not reported; PPV—positive predictive value; SCC—squamous cell carcinoma; TDS—teledermoscopy; 2WW—two-week wait.

**Table 7:** Implementation levers and pathway scale.

Study (Year)	Imaging Capture in Referral	Dermatologist Turnaround	Pathway Scale (Local→National)	Referral Channel/Platform	Notes/Equity Signal
May 2008 (UK) [20]	Clinical photos (store-and-forward)	NR	Local (single service)	Photo attachments to dermatology	Shorter time to clinic (14 vs. 24 d) without breaching 62-day target for nearly all melanomas
Ferrándiz 2012 (Spain) [17]	TD images from primary care to melanoma clinic	NR	Regional network	Store-and-forward TD	TD arm showed thinner melanomas and higher in situ/T1
Börve 2013 (Sweden) [18]	Smartphone teledermoscopy (macro + dermoscopy)	Often <24 h (feasibility context)	Multicenter (20 PHCs + 2 hospitals)	App-based capture with dermoscope	Feasibility established for GP-captured dermoscopy
Congalton 2015 (New Zealand) [19]	Teledermoscopy (macro + dermoscopy)	NR	Regional “Virtual Lesion Clinic”	Store-and-forward to dermatologist triage	Successful melanoma triage in VLC model
Dahlén Gyllencreutz 2017 (Sweden) [21]	Teledermoscopy referrals	NR	County/region	Mobile TDS referral vs. paper	Higher diagnostic agreement; operational efficiency
Teague 2022 (New Zealand) [22]	Teledermoscopy triage	~1 d advice typical in service	Regional service	VLC workflow	PPV ≈ 49%; FN ≈ 1.2% at scale
Chiru 2022 (UK, 2WW) [25]	Virtual pre-assessment (photos/video)	Same-day–few days (2WW)	System pathway (2WW)	Virtual consult platform	Maintained breach times vs. FTF; SCC detection ↑
Gao 2023 (New Zealand) [23]	Store-and-forward TD	Median ≈ 1.02 d to advice	Single center/service	Secure TD portal	“Time to action” ~64.8 d indicates downstream capacity limits
Koop 2023 (Estonia) [24]	Teledermoscopy nationwide	Dermatologist read ≈ 1–2 d (service benchmarks)	National (4748 cases)	Country e-health TDS	Diagnostic/plan accuracy comparable to experimental settings
Bouton 2024 (France, RCT) [27]	GP smartphone photos emailed to dermatologist	Faster access to consult vs. usual	Multi-site randomized	Email/secure transfer	Accelerated dermatologist consultation; melanoma subset included
Jaklitsch 2024 (USA) [26]	TD (async/sync) first vs. in-person first	Faster eval and biopsy via TD	Single academic system	Mixed TD	TD group had thicker, more ulcerated tumors (selection)
Zazo 2024 (Sweden) [28]	Countywide teledermoscopy	NR	Countywide	Standardized TDS	Sensitivity 98.9% for melanoma triage; safety at scale

e-health—electronic health infrastructure; FTF—face-to-face; GP—general practitioner; NR—not reported; PPV—positive predictive value; SCC—squamous cell carcinoma; TD—teledermatology; TDS—teledermoscopy; VLC—Virtual Lesion Clinic; 2WW—two-week wait; PHC—Primary Healthcare Centre.

## Data Availability

No new data were created or analyzed in this study.
